# *Streptococcus intermedius* Causing Necrotizing Pneumonia in an Immune Competent Female: A Case Report and Literature Review

**DOI:** 10.1155/2016/7452161

**Published:** 2016-11-06

**Authors:** Faris Hannoodi, Israa Ali, Hussam Sabbagh, Sarwan Kumar

**Affiliations:** Crittenton Hospital, 1101 W. University Drive, 2 South, Rochester, MI 48307, USA

## Abstract

We report a case of a 52-year-old immunocompetent Caucasian female treated for necrotizing* Streptococcus intermedius* pneumonia and review available literature of similar cases. Our patient presented with respiratory failure and required hospitalization and treatment in the intensive care unit. Moreover, she required surgical drainage of right lung empyema as well as decortication and resection. The review of literature revealed three cases of* S. intermedius* pneumonia, one of which was a mortality. Comparison of the published cases showed a highly varied prehospital course and radiological presentations, with a symptomatic phase ranging from 10 days to five months. Radiological findings varied from an isolated pleural effusion to systemic disease with the presence of brain abscesses. Immunocompetence appears to correlate well with the overall prognosis. In addition, smoking appears to be an important risk factor for* S. intermedius* pneumonia. In 2 (50%) of cases, pleural fluid analysis identified* S. intermedius*. In contrast, no organism was found in our patient, necessitating the acquisition of lung tissue sample for the diagnosis. In conclusion, both medical and surgical management are necessary for effective treatment of* S. intermedius* pneumonia. The outcome of treatment is good in immunocompetent individuals.

## 1. Introduction

*Streptococcus intermedius* is part of the* Streptococcus anginosus* subgroup (formerly* Streptococcus milleri*) [1–4]. It is a Gram-positive, catalase negative coccus that is nonmotile and is a facultative anaerobe [5]. They are normally found as part of the oral cavity and the gastrointestinal tract [4, 6, 7]. Although bacteria of the* S. anginosus* group are known to cause abscesses and systemic infections,* S. intermedius* pneumonia is rare and there are very few reported cases. We report an interesting case of necrotizing pneumonia in an immunocompetent patient caused by* S. intermedius* and we also review published cases in the reported literature.

## 2. Case Summary

A 52-year-old immunocompetent Caucasian female with a past medical history of asthma, that is only treated with albuterol rescue inhaler, and who is also an ex-smoker was transferred from the urgent care clinic to the emergency department (ED). She presented with shortness of breath with minimal activity and low oxygen saturation. Her prior symptoms were coughing and sputum production (greenish, thick, and nonbloody) for 6 weeks. She had been treated for community acquired pneumonia as an outpatient by her primary care physician and in an urgent care clinic. She was initially treated with oral erythromycin for a week. Two weeks following that, she received a week's course of ciprofloxacin. Her symptoms, however, failed to improve.

**Figure 1:**
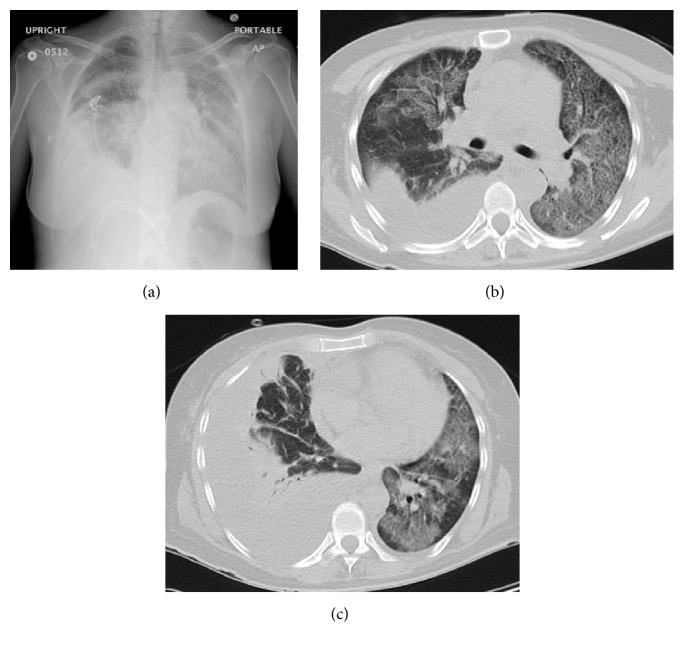
Chest X-ray (a) shows bilateral multilobar lung infiltrate with the appearance of loculated right pleural effusion. CT scan of the chest shows patchy airway disease throughout the upper lung lobes (b) as well as lower lung lobes (c).

Her vitals at presentation were temperature: 99.8°F, HR: 130 BPM, BP: 113/59 mmHg, RR: 36, and SpO2: 84% on room air. She was in severe respiratory distress and unable to complete a sentence without pausing or coughing. Lung exam findings included diminished breath sounds on the right lung base and rhonchi and crackles throughout both lungs on auscultation. ABGs showed pH 7.32, pCO_2_ 33, and pO_2_ 69 on 36% FiO_2_. The blood test results can be seen in Table 1, most significant of which is the leukocytosis. The extent of the pneumonic process is demonstrated in the images presented in Figure 1.

The patient was transferred to the intensive care unit and started empirically on aztreonam, vancomycin, and azithromycin with high flow oxygen. The patient's blood cultures, fungal cultures, legionella antigen and pleural fluid Gram staining, acid fast staining, and culture were all negative. Decortication, resection of the right upper lung lobe, and drainage of the empyema were performed on the third day of hospitalization and a chest tube was inserted.

Histology of the lung tissue sample showed acute and chronic pneumonitis with large areas of organization, focal abscess formation, and palisaded necrotizing granulomata. Special staining did not show any organisms. Culture of the tissue sample did grow* Streptococcus intermedius*, however. The organism was penicillin sensitive, but the patient has penicillin allergy. She was therefore given IV ceftriaxone, since beta-lactams have higher efficacy against streptococci than vancomycin. The patient was treated for a total of 14 days in hospital with IV antibiotics, of which, 7 days were in the intensive care unit.

## 3. Discussion

A search through PubMed and Google Scholar yielded only three reported cases of pneumonia caused by* S. intermedius*. All of the published cases are of male patients. 2 (67%) of the published cases are for patients in their 50s. Our case appears to be the only female patient. The immunocompetence of two of the reported cases is not mentioned. One case states the patient denies having risk factors for HIV [10]. Alcoholic liver cirrhosis is noted to be one of the conditions in one of the patients [9], which is a predisposing factor to infection as it leads to immune system dysfunction and relative immunoincompetence [11] (*refer to Table 2*).

The duration of symptoms for* S. intermedius* prior to hospitalization is highly variable, ranging from 10 days to 5 months. In one of the cases, the patient had empyema drained 4 months prior to presentation, though the responsible organism is not mentioned [8]. The radiological findings were also varied, ranging from presence of isolated pleural effusion to pneumonia with abscesses. This is consistent with the organism's property of causing local as well as systemic abscesses [6, 7].

As we can see in Table 2, there is one risk factor that most of the cases appear to share: a history of smoking. In general, smoking is a risk factor for pulmonary infections as it impairs ciliary function and increases mucous increases [12]. The patients appear to be hemodynamically stable on presentation, but they also have respiratory compromise and meet the criteria for a positive systemic inflammatory response. Fever is not always present even in severe respiratory disease as demonstrated in our case, neither is an elevated WBC as seen in one of the reported cases [10].

Treatment duration with antibiotics in our case and in one of the reported cases was 14 days, though in another it was 24 days [10].* S. intermedius* appears to be sensitive to beta-lactam antibiotics, though some cases of resistance have been reported [13]. In penicillin allergic patients, vancomycin is suggested as an alternative [14]. All the patients had a form of procedural intervention performed. This is expected as 3 (75%) of the patients had empyema with some form of loculation that necessitated drainage. Only one (25%) of the patients in the reported cases died from the pneumonia. This may be attributable to the concomitant presence of brain abscesses and liver cirrhosis [9].

As presented in Table 3, all of the cases that had a pleural fluid sample from the patient show it to be an exudate as LDH is >1000, meeting Light's criteria as well as the two-test and three-test rules [15, 16]. In both cases 1 and 3, organisms were identified in pleural fluid culture. In case 1,* S. intermedius* was identified following PCR and a homology search on the culture of the pleural fluid. In contrast, no organisms were identified in either pleural fluid microscopy or culture from the sample taken from our patient. The diagnosis was instead made by lung tissue sample culture. This shows that even when no organism is identified in the pleural fluid,* S. intermedius* can still be the etiologic agent. In both case 2 and in our case, histology of the lung tissue revealed necrotizing pneumonia, signifying the severity of disease caused by this organism (*refer to Table 3*).

## 4. Conclusion

To sum up,* Streptococcus intermedius* pneumonia has a wide-ranging prehospital incubation period, presentation, and radiological findings. Nonetheless, it is clear from both our case and the reported cases that* S. intermedius* causes severe disease that requires medical as well as surgical management to be treated effectively. Moreover, if pleural fluid microscopy and culture are negative, efforts should be made to obtain a lung tissue sample for microscopy and culture to identify the bacterium. Despite the severity of the disease,* S. intermedius* pneumonia shows good sensitivity to broad-spectrum antibiotics such as beta-lactams. As a result, patients have a good prognosis provided that they are immunocompetent.

## Figures and Tables

**Table 1:** Blood test results.

Glu	108	mg/dL
Na	142	mEq/L
K	3.0	mEq/L
Cl	108	mEq/L
HCO_3_^−^	14.5	mEq/L
AG	19.5	
BUN	27.0	mg/dL
Cr	1.1	mg/dL
GFR	52	
Lactate	1.9	mmol/L
WBC	29.2	10^3^/*μ*L
Neu	95	%
Lym	3.5	%
Mon	1.3	%
Eos	0.2	%
Hgb	11.4	g/dL
Plt	428	10^3^/*μ*L

AG: anion gap and GFR: glomerular filtration rate.

**Table 2:** Published cases of *Streptococcus intermedius *data.

Case	Our case	1 [8]	2 [9]	3 [10]
Age	52	79	55	52
Gender	F	M	M	M
Radiological diagnosis	Bilateral pneumonia, loculated right pleural effusion	Left upper lobe pneumonia and left pleural empyema	Right upper lobe pneumonia, bilateral brain abscesses	Loculated left pleural effusion
Duration of respiratory symptoms	6 weeks	—	10 days	5 months
Past medical history	Asthma	Surgical drainage of right empyema 4 months prior	Alcoholic liver cirrhosis	Hypertension, hyperlipidemia, and poor dental hygiene
Smoker status	Ex-smoker	Ex-smoker	—	Active smoker
Systolic blood pressure (mmHg)	113	104	—	125
Heart rate	110	118	—	93
Respiratory rate	36	—	—	—
Oxygen saturations	84% on room air	93% on 3 L	—	—
Temperature	99.8°F (37.7°C)	101.1°F (38.4°C)	101.3°F (38.5°C)	98.0°F (36.7°C)
Initial WBC	29.2	39.6	—	Normal (no number given)
Initial empiric antibiotics	Aztreonam, vancomycin + azithromycin	Meropenem	Ceftriaxone + ampicillin	Levofloxacin + clindamycin
Targeted antibiotics	Ceftriaxone	Meropenem	—	Levofloxacin
Total duration of antibiotics	14 days	14 days	—	24
Surgical intervention	Decortication, resection of right upper lung lobe and chest tube insertion	Left pleurectomy and chest tube insertion	Video-assisted thoracoscopic biopsy	Thoracocentesis and chest tube insertion
Outcome	Survived	Survived	Died	Survived

**Table 3:** Published cases of *Streptococcus intermedius* pleural fluid and tissue analysis.

Case	Our case	1 [8]	2 [9]	3 [10]
Total protein (g/dL)	4.0	4.3	—	4.2
LDH	1372	2873	—	6280
Glucose (mg/dL)	93	1.0	—	10
Gram staining	No organisms	No organisms	—	—
Pleural fluid culture	No growth	*S. anginosus group*	—	*S. intermedius*
Tissue histology	Necrotizing pneumonia, culture: *S. intermedius*	—	Necrotizing pneumonia, culture: *S. intermedius*	—

Case 2 did not have a pleural fluid sample; *S. anginosus*: *Streptococcus anginosus*.
